# To the Editors of the Medical and Physical Journal

**Published:** 1804-09-01

**Authors:** T. Trotter

**Affiliations:** Newcastle upon Tyne


### *To the Editors of the Medical and Phyfical Journal.*

GENTLEMEN,

ALTHOUGH I have not particularly attended to the Controversy, concerning the use of cold applications in cases of arthritic inflammation, in some late Numbers of your Journal, yet I presume the following facts will be acceptable to those gentlemen who have interested themselves in that discussion.

A few months ago, I was informed by a flag-officer of my acquaintance, that when in the service of Portugal, and employed in South America, he was seized with a severe fit of gout in the ball of the great toe; from which, by acute pain, and a considerable degree of inflammation, he was unable to move about. An old major of the army, who was subject to gout, had a command in the neighbourhood, and undertook to cure my friend, as he had often done himself, by immersing the foot in cold water. The river, in which the ship lay at anchor, happened to be much swoln with snow-water, that at a certain time of the year comes from the high mountains of that country; and in order that he might have full advantage of water at the coldest temperature that could be procured, he was carried in his barge to the middle of the river, and there plunged the affected foot into the stream. The water might probably be from 40° to 50° of Farenheit, which is cold for these latitudes. He felt immediate ease, and was soon relieved from this fit of gout. But while the old Major's method of cure was going on successfully with the English officer, he was affected himself with a painful fit of the same disease. He had recourse to his usual remedy. The pain and redness left the great toe, and he thought himself cured. But while he was exulting in his fortunate escape, good easy man! he was seized with apoplexy, and died in a few hours. Since that time, now thirty years ago, my friend has scarcely suffered any attack of regular gout; but he consulted me for complaints, which I conceive to be *anomalous* gout; such as vertigo, hemicrania, dimness of sight, and dyspepsia, with all its train of nervous affections. These he has had, in some degree or other, ever since he was in South America.

Now, I believe occurrences of this kind are not uncommon in medical practice; for we observe accidental cold produce all the evils that are here mentioned. The experience perience of physicians in all ages, has been very uniform in this respect. Hence the general opinion has wisely directed, that the gout should be consigned to *patience and flannel,* lest a more serious complaint should be the consequence of driving it from the extremities. I have not seen Dr. Kinglake's book on gout; but from what I may judge of his communications in your Journal, he will find it a difficult task to prove that this disease is a local one. One of the most exquisite cases of gout which ever came within my knowledge, was in a gentleman of thirty-two years of age. A hereditary disease was early brought into activity from intemperance. I have seen the inflammation of the extremities alternate with fits of epilepsy and hysteria, in the space of a few hours. I have seen him in the most excruciating pain from *gastrodynia, colica, and affections of the kidneys, ureters, and bladder,* when the redness of the joints suddenly disappeared; and I have stood by, while he took *opium, æther,* or *ardent spirit,* for relief, and the pain and redness would almost instantly be felt again in some of the articulations of the hand or foot. This gentleman died soon after.

I am, &c.

T. TROTTER.

*Newcastle upon Tyne, July* 20, 1804.

---

